# Antenatal and Perioperative Mechanisms of Global Neurological Injury in Congenital Heart Disease

**DOI:** 10.1007/s00246-020-02440-w

**Published:** 2020-12-29

**Authors:** Melinda Barkhuizen, Raul Abella, J. S. Hans Vles, Luc J. I. Zimmermann, Diego Gazzolo, Antonio W. D. Gavilanes

**Affiliations:** 1 Department of Pediatrics and Neonatology, Maastricht University Medical Center, Maastricht, The Netherlands; 2 School for Mental Health and Neuroscience, Maastricht University, Maastricht, The Netherlands; 3 Department of Pediatric Cardiac Surgery, University of Barcelona, Vall d’Hebron, Spain; 4 Department of Fetal, Maternal and Neonatal Health, C. Arrigo Children’s Hospital, Alessandria, Italy; 5 Instituto de Investigación e Innovación de Salud Integral, Facultad de Ciencias Médicas, Universidad Católica de Guayaquil, Guayaquil, Ecuador; 6 Department of Pediatrics, Maastricht University Medical Center, P. Debyelaan 25, 6229 HX Maastricht, The Netherlands

**Keywords:** Congenital heart disease, Hypoplastic left heart syndrome, Immaturity, Brain injury, Surgery

## Abstract

Congenital heart defects (CHD) is one of the most common types of birth defects. Thanks to advances in surgical techniques and intensive care, the majority of children with severe forms of CHD survive into adulthood. However, this increase in survival comes with a cost. CHD survivors have neurological functioning at the bottom of the normal range. A large spectrum of central nervous system dysmaturation leads to the deficits seen in critical CHD. The heart develops early during gestation, and CHD has a profound effect on fetal brain development for the remainder of gestation. Term infants with critical CHD are born with an immature brain, which is highly susceptible to hypoxic-ischemic injuries. Perioperative blood flow disturbances due to the CHD and the use of cardiopulmonary bypass or circulatory arrest during surgery cause additional neurological injuries. Innate patient factors, such as genetic syndromes and preterm birth, and postoperative complications play a larger role in neurological injury than perioperative factors. Strategies to reduce the disability burden in critical CHD survivors are urgently needed.

## Introduction

Congenital heart disease (CHD) is one of the most common types of birth defects. Moderate–severe forms of CHD occur in 0.6% of live births [1]. Over the past decades, survival has drastically improved. Currently, 90% of neonates with CHD survive into adulthood and, more adults are living with CHD than children born with CHD [2, 3]. However, strategies to reduce brain injuries and disability in critical CHD have been less successful. Although overt neurological dysfunction is rare in survivors of critical CHD, they have a typical neurodevelopmental impairment profile consisting of a mild reduction in cognitive performance, challenges with social interaction, inattention, impulsivity, impaired executive functions and worse pragmatic language [3–6]. CHD neurocognitive deficits are still present in young adulthood and likely persist into aging. This life-long neurocognitive disability presents a large burden to society [3, 6]. Here we provide a review of mechanisms of global neurological injury and neurodevelopmental delays in CHD. We will approach the matter by types of congenital heart disease, with specific mention to the most common pathologies, as well as and explore the factors that are known to impact neurodevelopment in these children.

The risk factors for neurocognitive impairment are multifactorial, interrelated and cumulative over time. These factors include: ((i) genetic and epigenetic factors, (ii) prematurity, (iii) socioeconomic factors, (iv) complications due to the heart disease resulting abnormal fetal cerebral blood flow and hypoxia–hyperoxia damage, (v) perioperative factors relating to cardiopulmonary bypass or deep hypothermic circulatory arrest, and (vi) postoperative factors [3, 5, 7, 8].

Most of these factors converge on brain maturity, additive injuries and hypoxia-reperfusion insult. The presence of CHD reduces the rate of fetal growth, particularly the rate of brain growth [9]. The developing brain is highly sensitive to oxygen balance changes. The white matter is particularly vulnerable to disruptions in cerebral blood flow because the blood vessels in the white matter have a lower vasodilatory response than blood vessels in the rest of the brain [10]. White matter injury (WMI) and focal infarctions of the gray matter are the most common injuries seen in infants with severe CHD [11]. Preoperative WMI is present in 16–39% of term infants with CHD, and 35%-67% of CHD infants develop new postoperative WMI. Preoperative WMI is particularly common in infants diagnosed with single-ventricle physiology, with aortic arch obstruction, and in infants with a low brain maturity score. Postoperative injury is associated with preoperative injury, the use of cardiopulmonary bypass (CPB) with regional cerebral perfusion, and lower cerebral hemoglobin oxygen saturation during the myocardial ischemic period. Excessive reoxygenation during CPB may also contribute to postoperative brain injury [4, 12–18].

Infants with hypoplastic left heart syndrome (HLHS) with a low birth weight have an overall poor growth rate, longer hospital stays and worse neurodevelopmental outcomes [19]. In infants with undergoing surgical repair for HLHS, d-TGA, truncus arteriosus and interrupted aortic arch with a ventricular septal defect, the presence of WMI further delays brain growth, which increases susceptibility to additional postoperative mild WMI [16, 20]. The majority of neonatal magnetic resonance imaging (MRI) lesions resolve within 4–6 months [18], but neurodevelopmental delays occur throughout life. At 1–1.5 years of age, infants that had a surgical repair for CHD had a lower scores on both the Psychomotor Development Index (PDI) and Mental Development Indexes (MDI) of the Bayley Scales of Infant Development 2nd edition, and the Griffiths developmental scales and were more likely to have neurological abnormalities [21, 22].

Neurodevelopmental delays are associated with reduced growth in specific brain regions. Neonates and infants with CHD have smaller brain volumes in the frontal lobe, parietal lobe, cerebral white matter, subcortical gray matter, cerebellum and brainstem [9, 23, 24]. In newborns with cyanotic CHD, reduced subcortical gray matter and increased CSF volumes associated with poor behavioral state regulation, while in newborns with non-cyanotic CHD behavioral state regulation deficits were associated with reduced cerebellar volumes [24]. Around 1 year, infants with biventricular CHD still have lower cerebral white matter, cerebellar white matter and brain stem volumes. Reduced cerebral white matter volume correlated with lower language scores [25]. Reduced perioperative brain growth of specific cortical regions, such as the Heschl’s gyrus and anterior planum temporale in the left temporal plane, also associated with language score deficits at 1 year of age [26]. Postoperative WMI is associated with lower IQ scores and attention deficits at school age [17]. By adolescence, the neurodevelopmental delay persisted and manifested as impairments of neuromotor, intellectual, executive function, visuomotor, perception and integration skills. Lasting cerebral WMI and microstructural alterations, cerebellar white matter and cerebellar volume loss contributed to the neurodevelopmental deficits [27–30].

## Types of CHD

CHD is a diverse group of disorders classified by disease severity and cyanosis. Severe cyanotic CHD syndromes include d-transposition of the great arteries (d-TGA), tetralogy of Fallot (TOF), double outlet right ventricle, truncus arteriosus and single-ventricle disorders. Single-ventricle physiology is divided into hypoplastic left heart syndromes (HLHS) with aortic or mitral atresia, and hypoplastic right heart syndromes with tricuspid or pulmonary atresia. Severe forms of non-cyanotic heart disease include atrioventricular septal defects, ventral septal defects, patent ductus arteriosus, aortic and pulmonary stenosis [1].

### Cyanotic Heart Diseases

#### Dextro-Transposition of the Great Arteries

d-TGA is a cyanotic heart disease that occurs when the two main arteries carrying blood away from the heart are reversed. During d-TGA, oxygen-rich blood reaches the pulmonary vasculature and oxygen-poor blood is pumped to the brain and body. The l-TGA form preserves the flow of oxygen-rich blood around the body and is less severe than d-TGA [6, 7, 28, 31]. Preoperative brain injuries are common in d-TGA. Fetal injuries occur in around 16%, preoperative brain injuries, including WMI, stroke and intraventricular hemorrhages occur in 30–41% and postoperative WMI in 25% of neonates studied [12, 32, 33]. At birth, infants with d-TGA have a smaller head circumference and a tendency to have an overall reduced brain volume [34–36].

Adolescents with d-TGA have abnormal WM microstructure and reduced gray matter volumes and thicknesses in the parietal, midline, and subcortical brain regions, which correlates with their neurocognitive performance [31, 37, 38]. They typically have lower test scores within the average range for academic achievement, memory, executive functions, visual-spatial skills, attention, and social cognition than a normative population. Up to 65% of adolescents with d-TGA has received remedial academic or behavioral services [7, 28].

#### Single-Ventricle Physiology

Single-ventricle physiology occurs when one of the heart ventricles is smaller, underdeveloped or missing a valve. Because there is only one functioning ventricle, the same blood flows to the lungs and the body. Left-sided single-ventricle defects include hypoplastic left heart syndrome (HLHS) where the entire left side of the heart (aorta, aortic valve, left ventricle and mitral valve) are underdeveloped, mitral valve atresia which impairs blood flow from the left atrium to the left ventricle, and aortic arch obstructions that impair blood flow from the left ventricle to the body. Right side lesions include hypoplastic right heart syndrome, tricuspid atresia where the blood cannot flow from the right atrium to the right ventricle, and pulmonary atresia where blood cannot flow from the right ventricle to the lungs [1, 6]. Somatic growth restriction occurs in approximately 16% of fetuses with HLHS. Some HLHS fetuses have a small head size and WMI at mid-gestation, but the majority of fetuses have a normal head size. During the second and third trimester, approximately 50% of fetuses subsequently develop poor head growth that manifests as the reduced head circumference. Microcephaly occurs in 12% of HLHS newborns. Microcephaly was associated with a low birth weight and birth length, but not with the aortic valve anatomy [5, 34, 39, 40].

Autopsy analysis of 11 HLHS fetuses showed some degree of chronic WMI in all the fetuses, despite normal brain weights. The WMI localized to the deep and intermediate white matter, with widespread multifocal involvement of adjacent central nervous system (CNS) structures and gray matter injury. None of the fetuses had infarcts [39]. A second autopsy analysis of 40 HLHS infants found that 55% of the cohort had no acquired brain lesions, and the remaining 45% had hypoxic-ischemic lesions or intracranial hemorrhage [41].

MRI studies have identified preoperative WMI or stroke in 16–45%, and postoperative WMI or strokes in 50% of neonates with HLHS [12, 32, 33]. Brain lesions occur in up to 75% of neonates with tricuspid atresia [33]. Single-ventricle physiology delays cortical growth and gyrification [42].

HLHS delays performance on the PDI to a larger extent than other types of single-ventricle deficits [43]. At around 14 months of age, neonates that had corrective surgery for HLHS had reduced PDI and MDI scores. This developmental delay was more highly associated with innate patient factors, like low birth weight, maternal education and genetic anomalies, and with complications after the surgery during the first year of life, than with intraoperative management strategies [44]. The PDI and MDI delays in HLHS persist at 30 months of age [43].

Adolescents with single-ventricle disease have widespread WM microstructure abnormalities, reduced gray matter volumes, and asymmetric brain sulcal patterns on MRI. They also have compromised integrity of multiple cortical, subcortical and cerebellar regions that are involved in autonomic control, mood and cognition. Adolescents with single-ventricle disease have deficits in all of these functional areas [45–47]. Volume loss of the mammillary bodies in the limbic system associates cognitive and memory deficits [48]. The majority of school-aged HLHS survivors have IQ scores within the normal range, but a lower mean performance than the general population and a third of the children require special education [49, 50]. Children and adolescents with HLHS also have impaired executive functioning and intellectual disability occurs in 18% of HLHS survivors [28, 49]. Attention deficit hyperactivity disorder (ADHD) occurs in 28–50% of school-aged survivors, gross motor deficits such as cerebral palsy occur in 17–64%, clinically significant anxiety occurs in 18%, and approximately 50% of HLHS survivors use remedial education [10, 51].

#### Tetralogy of Fallot

Tetralogy of Fallot (TOF) is a severe cyanotic heart disease that consists of four congenital anomalies: (i) a right ventricular outflow tract obstruction, (ii) a ventricular septal defect, (iii) an overriding aorta which receives blood from both ventricles, and (iv) right ventricular hypertrophy [52]. Currently, the 20–30-year survival rates of TOF is close to 90%, but their survival rates decrease after middle age and these patients have life-long morbidity [53]. TOF affects brain growth from early in gestation. Fetuses with TOF already have reduced cortical and subcortical gray and white matter volumes and enlarged CSF spaces by 25 weeks of gestation, which persist throughout gestation. The mean cerebellar volume was not affected by TOF [52]. At birth, neonates with TOF have reduced head circumference [34]. Around 1/5th of fetuses and neonates with TOF also have abnormal preoperative MRIs [33]. During adolescence, patients that had surgery for TOF had lower scores on tests of academic achievement, memory, executive functions, visual-spatial skills, attention and social cognition. Co-morbid genetic abnormalities occur in up to 25% of adolescents, which further impairs neurocognitive performance. In TOF without other genetic abnormalities, the strongest predictors of adverse neurocognitive outcomes were related to surgical complications and postoperative seizures [54]. TOF also increases the incidence of motor dysfunction, which correlates with neurological dysfunction, lower IQ scores, reduced expressive language, behavioral problems, reduced expressive language, self-esteem and school competence around 7 years of school age [55, 56].

### Non-cyanotic Disorders

#### Aortic Valve Stenosis and Atrial, Ventricular and Atrioventricular Septal Defects

There are fewer studies on outcomes after non-cyanotic CHD. Somatic growth restrictions occur in around 13% of neonates with isolated aortic valve stenosis. Aortic valve stenosis affects antegrade aortic blood flow, which may reduce cerebral blood flow and impact CNS development. Aortic valve stenosis leads to microcephaly or head growth restriction in approximately 25% of neonates, despite relatively mild blood flow disturbances. The reduction in head growth is likely due to complex factors which affect both CNS and cardiac development, rather than due to reduced antegrade aortic blood flow [39].

A study of a mixed group of children with predominantly non-cyanotic CHD that had open-heart surgery at school age found no deficits in general intelligence and neurocognitive functioning before and after surgery. However, this study excluded 26% of children that had a physical or mental deficit before surgery from their analysis [57]. A second study of surgical correction in children with non-cyanotic CHD also did not find any baseline differences on any neuropsychological domain before surgery, and a mild postoperative decline in cognitive function at 6 months after surgery. This decline was not specifically attributable to using CPB during the surgery [58]. Studies of children with atrial septal defects found mean scores for IQ, general conceptual ability (analogous to IQ), academic achievement, language and memory within the normal range before and after correction with surgical repair or a catheter-delivered device. These children were also generally healthy. However surgical correction affected visuospatial and visuomotor skills, which reduced their performance IQ scores in one study [59, 60].

The incidence of preoperative MRI abnormalities in ventricular septal defects (VSD) and atrioventricular septal defects (AVSD) ranges from 16–50%, but relatively low amounts of infants have been studied [33]. At 1 year, the MDI and PDI scores of children that underwent surgical repair for a VSD with or without an additional aortic arch obstruction were within normal limits [61]. Around 6–7 years of age (range 3–16 years), children that had surgical correction of VSD with or without cardiac insufficiency had intermediate neurodevelopmental deficits. Children without cyanosis before the operation had a normal performance on most domains, including full-scale IQ, internalizing behavior, externalizing behavior, executive functioning, quality of life, verbal memory, attention and concentration, cognitive flexibility and problem-solving, and academic skills. However, children with VSD had reduced performance IQ, visual-motor ability and reduced adaptive behavior deficits. Another study of VSD complicated by cardiac insufficiency at the time of operation found that around 7 years of age these children had increased internalizing and externalizing problems, reduced school performance and lower use of expressive and receptive language, comparable to children with TOF. Therefore the degree of cardiac insufficiency affects neurodevelopmental outcomes in children with surgically corrected VSD [55, 56, 62]. Around 9 years of age, children with surgically corrected VSD had subtle problems in attention and visuospatial information processing, while children with surgically corrected atrial septal defect secundum type had lower scores on tests of visuospatial processing, working memory, sensorimotor functioning, language, attention, and social perception and lower school performance [63, 64].

#### Patent Ductus Arteriosus (PDA)

The ductus arteriosus is a fetal blood vessel between the aorta and the pulmonary artery that normally closes shortly after birth. Sustained opening of the ductus arteriosus or PDA may lead to heart failure later in life. PDA is one of the most common types of CHD with an incidence between 5 and 10% in term infants, and up to 60% in preterm infants born before 29 weeks of gestation. In term infants, PDA is associated with functional defects, while in preterm infants, PDA is associated with prematurity. PDA is commonly associated with morbidity and mortality in preterm infants and often accompanied by other complications of prematurity, such as necrotizing enterocolitis, intraventricular hemorrhage, pulmonary hemorrhage, chronic lung diseases and retinopathy of prematurity [65–69].

PDA reduces cerebral oxygen saturation. WM and cerebral injuries due to immaturity and hypoxic-ischemic injuries are also common in preterm PDA and a major cause of neurodevelopmental impairments. Treatment options for PDA consist of pharmacotherapy with cyclooxygenase inhibitors, fluid restriction, or surgical ligation. Approximately 20–36% of preterm survivors of PDA have a neurodevelopmental disability such as cerebral palsy, hearing loss, blindness, or PDI and MDI scores below 70 at 1.5–2 years of age. Infants that had a surgical ligation had a higher incidence of neurodevelopmental deficits than infants treated pharmacologically or left untreated. However, the higher incidence of neurodevelopmental disorders after ligation treatment may be due to confounding by additional risk factors, rather than the ligation itself. At birth, risk factors for neurodevelopmental disability include gestational age, birth weight, multiple gestation, antenatal corticosteroids, intrauterine growth restriction and sex. At the time of discharge, neonatal morbidities of prematurity, sepsis and major brain injury are associated with neurodevelopmental impairments at 18–24 months of age [65–69]. Since brain injury and neurodevelopmental disability in PDA is intertwined with the complications of preterm birth in general, we excluded PDA from the rest of the review.

## Factors that Predict Neurological Injury and Neurodevelopmental Deficits

### Preoperative Factors

Patient-specific preoperative factors are stronger predictors of adverse neurodevelopmental outcome than intraoperative factors [10, 22, 70, 71].

#### Delayed Brain Growth

A substantial amount of neurological damage in critical CHD occurs prenatally due to global CNS dysmaturation of white and gray matter, resulting in impaired neuronal connectivity and long-term neurodevelopmental impairment. Human brain development begins at the 3^rd^ week at gestation with the differentiation of neural progenitor cells and continues into late adolescence. Neurogenesis starts at the 6th week of gestation and is mostly complete by mid-gestation [72]. The fetal heart development is nearly complete by the 7th week of gestation and the occurrence of critical CHD can disrupt fetal blood flow and oxygen delivery to the brain for the rest of the gestational period [73]. By the end of the 8th week of gestation, the basic structures of the CNS are established. The subsequent period until mid-gestation is critical for the formation of the neocortex. Around mid-gestation, the myelination process starts. During the third-trimester blood supply to the brain increases to 25% of the combined ventricular output to allow the increased formation of synaptic connections and neuronal activity. By the end of gestation, the formation of major subcortical pathways like the thalamocortical pathway is complete. While the production and migration of neurons is largely completed prenatally, the proliferation and migration of glial precursors and differentiation into astrocytes and myelinating oligodendrocytes continue after birth and the maturation of glial cells continue into early childhood. These processes play a critical role in the functional maturation of neural circuits. Substantial cortical neurogenesis and migration of neurons to the frontal lobe also occur postnatally [8, 72, 74]. Critical CHD impairs cortical growth and maturation by reducing the proliferation of neural progenitor cells and neurogenesis in the subventricular zone [75]. The brain size only reaches 90% of the adult brain volume by age 6 [72].

Pregnancies in which the fetus has a CHD often have an impaired uteroplacental environment and increased incidences of pre-eclampsia, preterm birth and low gestational age births. Impaired fetal-placental oxygen exchange reduces systemic and cerebral oxygenation that results in a delay of body and brain growth [36, 76, 77]. The majority of studies of CHD, particularly studies of HLHS, d-TGA and TOF, report impaired fetal brain growth [34]. Structural brain abnormalities are present in 28% of fetuses with CHD. The most common abnormalities are enlarged ventricles, agenesis of the corpus callosum, ventricular bleeding, increased extra-axial space, vermian hypoplasia, white matter abnormalities and delayed brain development [78].

At birth, infants with CHD typically have a birth weight and birth length below average, a mean head circumference 1 standard deviation below normal, and a delay of 1 month in structural brain development. Neonates with CHD have delayed gyrification, a 21% reduction in overall brain volume and regional brain volume reductions of 8–28% [9, 12, 19, 32, 34, 79, 80]. In particular, smaller brain volumes in the frontal lobe, parietal lobe, cerebral white matter, thalamus, cerebellum and brainstem have been found around the time of birth. The reduction in frontal and parietal lobe size correlates with delayed white matter microstructure development, while reduced subcortical and cerebellar volumes associates with regional deficits in cerebral WM energy metabolism [9, 23].

The underdeveloped brain metabolism and WM microstructure of term infants with CHD increase both the risk of preoperative and postoperative WMI and the severity of the brain injury [12–14, 16, 81]. WMI occurs in the central, frontal and posterior periventricular regions in both term and preterm CHD infants [12–14]. Neonatal brain injuries are already detectable on a fetal MRI in 27% of cases [33], thus brain injuries in this population are a mixture of prenatal, perinatal and postoperative injuries. Preoperative brain injury in term neonates is associated with subsequently impaired corticospinal tract development [82]. Delays in brain growth vary by the type of CHD. Neonates with HLHS have a lower global and regional brain growth rate than neonates with d-TGA [20].

Abnormal brain growth and brain injury in the antenatal period has life-long effects. Focal WM lesions consisting of small punctate mineralization or WM iron deposits are relatively common on structural MRIs of adolescents with CHD. These MRI abnormalities are likely related to micro hemorrhage that occurred at the time of corrective surgery [7, 54]. When compared to controls, adolescents with CHD have lower total brain volumes and lower volumes of WM and cortical, subcortical and cerebellar gray matter but comparable cerebrospinal fluid (CSF) volumes. Adolescents with cyanotic CHD have more WM and subcortical brain volume loss than non-cyanotic CHD. The reduction in regional brain volumes correlated with impairments in cognitive, motor and executive functions [83]. After controlling for the total brain volume, the extent of hippocampal volume loss after CHD correlated with total IQ, working memory, episodic memory and verbal comprehension. The extent of WM volume loss correlated with verbal comprehension and motor performance and the cerebellar volume correlated with working memory and static balance [83–85]. Adolescents with CHD also have global WM microstructural abnormalities and a disruption of the WM network topology. Some of these WM disturbances correlate with performance in specific functional domains.

On a microstructural level, adolescents have a lower apparent density of axonal packing, but not altered axonal orientation. The reduced WM axonal packing could impair communication between different brain regions [86]. The degree of global WM network topology disturbances correlates with overall neurocognitive performance, WMI in the uncinate fasciculus correlates with verbal memory impairments, WMI in the frontal lobe associates with working memory deficits, and WMI of the middle cerebellar peduncle correlates with auditory attention span [30, 31, 87].

Fetuses with CHD have lower cerebellar volumes and an overall reduced growth rate of the cortical and subcortical gray matter and cerebellum over the fetal period, which results in reduced total brain volumes at birth and 3 months of age [80, 88]. CHD reduces the rate of brain volume gain over the third trimester. Fetuses with CHDs with single-ventricle physiology experience a greater slowing of brain growth than fetuses with two-ventricle CHDs. Fetuses with two-ventricle CHD have a steady increase in brain volume across most regions during the third trimester. In fetuses with single-ventricle CHD the rate of cerebral brain growth slows between 32 and 35 weeks of gestation, the rate of total brain, cortical plate, and deep gray matter growth slows around 35 weeks of gestation, and cerebellar growth slows around 34 weeks of gestation. Fetuses with single-ventricle and two-ventricle CHD had similar rates of WM volume gain over the third trimester [89]. There is a strong correlation between the total brain volume and volumes of the cortical gray matter, unmyelinated white matter and CSF on the fetal MRI at 33 weeks of gestation and neonatal MRI within the first week after birth [90].

#### Cerebral Blood Flow Obstructions

Fetuses have different blood flow patterns than neonates because their gas exchange occurs in the placenta. The oxygenated blood from the placenta flows from the right atrium across the foramen ovale to the left atrium, which streams oxygen-rich blood to the brain. Critical CHD causes modifications to the intracardiac circulation, which changes the characteristics of cerebral blood flow to the brain. CHD reduces both the blood flow to the brain (ischemia) and the oxygen content of the blood (hypoxia). Inadequate blood flow to the brain reduces both oxygen and glucose delivery, which affects brain growth. The fetus attempts to compensate by reducing cerebral vascular resistance to increase cerebral blood flow, but this ‘brain sparing’ mechanism is insufficient to ensure adequate blood supply to the brain [8, 91].

The type of CHD affects fetal cerebrovascular blood flow distribution and the level of brain immaturity. Single-ventricle physiology occurs when one of the ventricles is smaller, underdeveloped or missing a valve. In HLHS, the left side of the heart is underdeveloped. Oxygen-rich blood from the placenta mixes with deoxygenated blood in the right atrium, which reduces the overall oxygen content of the blood. Blood with a reduced oxygen content is ejected into the pulmonary trunk and the ductus arteriosus. Retrograde blood flow and a reduced diameter of the aortic arch further restricts blood flow into the brain. In HLHS, the fetal brain thus receives less blood (ischemia) and the blood that flows to the brain has a reduced oxygen content (hypoxia). In fetuses with left-ventricular outflow tract obstructions, the flow of blood to the brain is reduced (ischemia) but the relative oxygen content of the blood that reaches the brain is within normal limits. The brain compensates for left-sided lesions by lowering cerebrovascular resistance to increase blood flow to the brain. Fetuses with obstructions of the right side of the heart have higher cerebrovascular resistance than infants with HLHS. In d-TGA, the two main arteries carrying blood away from the heart are reversed so that the brain receives relatively deoxygenated blood from the superior vena cava through the right ventricle. Thus d-TGA causes a severe reduction in the oxygen content of the blood delivered to the brain (hypoxia), but less cerebral blood flow disturbances (ischemia) than left-ventricular outflow tract obstructions and single-ventricle physiology with aortic arch obstructions [8, 36, 42, 81]. These differences in cerebrovascular blood flow distribution likely affect subsequent brain injuries [8, 10, 35, 92].

Fetuses with HLHS have a progressive delay white and gray matter brain growth, brain metabolism and cortical folding over the third trimester [93, 94]. The delays in WM microstructural development and WMI are worse in infants with smaller ascending aortas or aortic atresia [95, 96]. In severe HLHS, there is a decrease in cerebral oxygen saturation and cerebral oxygen extraction accompanied by a switch to anaerobic metabolism starting in the fetal period. More than 50% of neonates with HLHS have elevated brain lactate levels, sometimes accompanied by focal ischemic lesions [18, 94, 97]. The relative hypoxia persists throughout early childhood. Infants with single-ventricle physiology (including HLHS) have reduced blood oxygen saturation at 1 and 3 years of age [98]. Reduced cerebral oxygen saturation persists in HLHS after stage I Norwood RVTA shunt and stage II Glenn repairs, but returns to normal after the stage III Fontan repair [97].

Fetuses with d-TGA have impaired brain growth and metabolism over the third trimester [94]. However, the impairment in brain growth in d-TGA is less severe than in single-ventricle physiology with aortic arch obstruction. Neonates with d-TGA or a left ventricle outflow tract obstruction have more mature WM microstructure than infants with single-ventricle physiology with aortic arch obstruction [81].

CHD delays cortical growth and maturation: infants with mild forms of d-TGA have normal brain development of gyrification, while gyrification is delayed in infants with single-ventricle physiology. However, infants with severe d-TGA, that requires preoperative palliation with balloon atrioseptostomy, have a higher risk of delayed gyrification and reduced cortical volumes [42]. The use of balloon atrioseptostomy also increases the risk of WMI [99]. At 1 year, both infants with d-TGA and single-ventricle physiology have reduced brain volumes. But by 3 years of age onwards, brain volumes in the d-TGA group normalize but remain small in infants with the single-ventricle disease [98]. Severe hypoxia and acidosis before corrective surgery in d-TGA predispose to neurodevelopmental impairments during adolescence [100].

The impact of other types of CHD on blood flow and cerebral oxygenation is varied. TOF, patent ductus arteriosus and pulmonary atresia reduce cerebral oxygen saturation. However, children with ventricular septal defects and aortic coarctation have cerebral oxygen saturation levels comparable to controls [97].

#### Prenatal Diagnosis

The majority of CHD cases can be diagnosed with prenatal echocardiography. Tertiary perinatal centers can detect 85–95% of CHD cases prenatally, but the average prenatal detection rate in the US is around 34%. CHD lesions visible on a 4 chamber view are more likely to be detected prenatally than those requiring outflow tract visualization [101, 102].

Prenatal treatments of fetal abnormalities are a rapidly developing area. However, prenatal detection of the CHD and brain injuries do not necessarily improve outcomes. Most diagnostic techniques can only detect fetal heart defects after 18 weeks of gestation, by which time considerable organ damage has already occurred which can no longer be corrected, even with intrauterine surgery [102].

In CHD infants with standard mortality risk, a prenatal diagnosis reduces the risk of death due to cardiovascular compromise before the planned surgical intervention but does not affect mortality after surgery. However, the most critical infants may be considered too high risk for surgery and only receive palliative care and pregnancies with a prenatal diagnosis of complex CHD may be terminated. Prenatal diagnosis also increases the likelihood of a scheduled delivery, which results in infants being born earlier with lower birth weights [103–107].

Despite the potential drawbacks of prenatal diagnosis, in neonates with single-ventricle physiology or d-TGA, a prenatal diagnosis reduces the incidence of perioperative brain injuries and increases the rate of WM microstructural development. These beneficial brain findings are likely due to improved cardiovascular stability [107]. The brain volumes on fetal MRI late in gestation strongly correlate with neonatal brain volumes in CHD. Fetuses with lower brain volumes on MRI are at a higher risk of having a neonatal ischemic brain injury, and neonatal brain injuries are already detectable on a fetal MRI in 27% of cases [33, 90]. Therefore, a fetal diagnosis of CHD is considered beneficial in terms of preoperative mortality and brain development. Once prenatal investigations confirmed the diagnosis of an operable CHD the main target for physicians will consist of an early optimized hemodynamic postnatal stabilization that in turn will improve neurological morbidity and WM development [6, 108, 109].

#### Other Preoperative Factors

Around 30% of infants with CHD have an additional genetic anomaly. Infants with an additional genetic anomaly are born at a lower gestational age and weight, have a smaller head circumference at birth and worse performance on the PDI and MDI at 1 year of age [70, 110].

In patients without genetic anomalies, innate risk factors (birth weight, Apgar score, race, APOE-E genotype) are associated with lower scores on the PDI. Low birth weight, Apgar score, male sex, maternal education level, genetic anomalies and APOE-E genotype are associated with lower scores on the MDI between 1 and 1.5 years of age [22, 70, 110]. Interestingly, the APOE-E2 genotype is associated with worse neurodevelopmental outcomes in CHD, while the APOE-E2 genotype is protective against the late-life neurodegenerative disease Alzheimer’s disease [110, 111]. Male sex, delivery by C-section, and an acute hypoxic insult due to preoperative cardiac arrest are also risk factors for WMI in CHD [6, 96, 99]. Male sex is also a risk factor for worse outcomes after a hypoxic-ischemic insult in otherwise healthy newborns [112]. Preoperative seizures predicted lower IQ in school age children with HLHS [49].

### Perioperative Factors

The majority of neonates with mild-severe CHD requires surgery within the first few weeks after birth to survive. Intraoperative brain injuries are predominantly due to the methods of CPB and deep hypothermic circulatory arrest (DHCA) used after stopping blood circulation and brain function for surgery. Complications of these methods include low cardiac output syndrome, hypoperfusion, air emboli and systemic inflammation [6, 73, 113].

#### Embolism

Most heart surgery procedures involve opening the left side of the circulation, which brings a risk of vascular air embolism, hypoperfusion and thromboembolism. The risk of stroke in CHD has been reduced over the last few decades, but preoperative strokes still occur in 2–25% and postoperative strokes occur in 0–19% of infants with surgery for CHD [113–115].

#### Choice of Surgical Procedures

##### The Norwood Procedure for HLHS

The stage I Norwood procedure is the first of three palliative operations for HLHS. While the stage II (Hemi-Fontan or bidirectional Glenn) and stage III Fontan procedures are now relatively low risk, the Norwood procedure remains a high-risk procedure [116, 117]. Cerebral oxygen saturation in HLHS remains low after the stage I and II repairs but improves after the stage III Fontan procedure [97]. Altered cardiovascular hemodynamics after stage II repair, particularly increased atrial filling and Glenn pressures, associated with reduced brain volumes and worse neurodevelopmental outcomes [118]. Both survival and neurodevelopmental outcomes after the Norwood procedure have improved between 1998 and 2005, but neurodevelopmental scores of survivors remain below normal [119]. However, neurodevelopmental outcomes in HLHS survivors is comparable between infants that had a staged reconstruction with the Norwood procedure and infants that had a heart transplant, which indicates intrinsic patient factors play a larger role than intraoperative factors. Preoperative seizures and a longer total DHCA time predicted lower IQ scores in HLHS survivors [10]. Hemodynamic instability and postoperative death after the Norwood procedure remain problematic. Here we discuss several variations on the Norwood procedure. The first variable that can influence the surgical outcome is the choice of shunt used during the operation. Several studies have compared outcomes after the modified Blalock–Taussig shunt (MBTS) to the right ventricle-to-pulmonary artery (RVPA) shunt modification of the Norwood procedure. The use of the RVPA shunt has reduced mortality rates and improved the PDI score of infants with HLHS, likely because it promotes a more stable hemodynamic status [19, 119, 120]. However, the RVPA shunt may cause fibrotic tissue to develop over the long-term and an individualized shunt choice based on birth weight and aorta size can further improve outcomes [117].

A second consideration is the use of support techniques during the operation. Regional cerebral perfusion (RCP), also known as antegrade or low-flow cerebral perfusion, is a support technique that provides antegrade cerebral blood flow and maintains oxygen supply to the brain, while still maintaining a relatively bloodless intraoperative field. This allows more time to complete the repair [6, 121]. The uptake of RCP has increased in recent years [119].

A single randomized clinical trial did not find improved neurodevelopmental outcomes of RCP vs. deep hypothermic circulatory arrest (DHCA) used in the Norwood procedure at 1 year of age or during a follow-up investigation at 5–8 years [122, 123]. However, further improvements of the RCP technique have been suggested including neurological monitoring, with near-infrared spectroscopy (NIRS) and transcranial Doppler ultrasound (TCD), to adjust flow rates during RCP to standardize cerebral oxygen delivery for the individual patient. These results in an increased cerebral flow rate, which does not appear to cause more neurological injuries than the standard CPB [16, 121, 124]. Standardizing cerebral oxygen flow during RCP has improved the cognitive outcomes of survivors to levels comparable to the reference population at 1 year of age. However, mean language and motor outcomes remain lower than the reference population mean by 0.8–0.9 standard deviations. The duration of RCP was not associated with adverse outcomes and prolonged RCP appears to be safe [121]. The age-at-surgery is a remaining consideration. A shorter time to surgery may decrease perioperative WMI [125].

##### Arterial Switch Operation for d-TGA

The arterial switch operation (ASO) is the standard of care for neonates with d-TGA. The procedure has a low mortality rate. Lower cerebral oxygen saturation before and during the procedure, longer CPB and cross-clamping times, and anesthetic use were associated with adverse neurodevelopmental outcomes [100, 126, 127].

#### Cardiopulmonary Bypass

Open-heart surgery with CPB is standard care for CHD. However, open-heart surgery causes a planned ischemia–reperfusion injury, which resembles the hypoxic-ischemic injury during perinatal asphyxia [113]. CPB is associated with cerebral microhemorrhages, which are still present in early adulthood (mean age 26.7 years) [128]

The use of CPB with RCP and lower cerebral hemoglobin oxygen saturation during surgery increases the risk of WMI and adverse neurodevelopmental outcomes [4, 126]. Lower nasopharyngeal temperatures during CPB were associated with a lower score on the PDI and a lower head circumference at 1–1.5 years of age [70]. Longer CPB times associated with adverse neurodevelopmental outcomes. However, the predictive value of CPB time was lower than that of preoperative and postoperative factors. A longer CPB time is likely a surrogate marker for more complex surgery with longer postoperative stays [49, 71].

CPB techniques are continuously improving. Notable advances in CPB technique over the last few years include the use of low prime CPB circuits and modified ultrafiltration [119]. A recent systematic review of interventions during CPB found that the clinical evidence for neuroprotective and neuromonitoring interventions curing CPB was limited and the only intervention with sufficient evidence was avoiding extreme hemodilution during CPB [129]. Low prime CPB circuits minimize hemodilution that improves the hematocrit values and reduces the need for red blood cell transfusions [119, 130, 131]. Modified ultrafiltration reduces systemic inflammation during CPB, which reduces morbidity and mortality [132].

#### Cardiopulmonary Bypass Complications

##### Low Cardiac Output Syndrome

Low cardiac output syndrome (LCOS) is a major perioperative complication after open-heart surgery with CPB. LCOS has multiple contributing factors, including ischemia–reperfusion injury, surgical trauma, endothelial dysfunction and the activation of inflammatory, complement and coagulation processes [113, 133]. Preoperative multifocal brain injuries are also associated with LCOS [134]. CPB affects cerebrovascular resistance by increasing the pulsatility of the middle cerebral artery during surgery [133]. Increased cerebrovascular resistance, elevated venous pressure, LCOS and low blood pressure compromise oxygen delivery to the brain, which increases the risk of postoperative neurological damage [113]. LCOS contributed to 30% of new postoperative multifocal brain injuries in a clinical cohort [134]. Several putative biomarkers of neurological damage during CPB have been identified. Infants with postoperative neurological damage have higher plasma levels of the brain injury protein activin A during CPB [135]. The plasma levels adrenomedullin, a vasoactive peptide that regulates cerebral blood flow, decreases during cardiac surgery with CPB. Low adrenomedullin levels at the end of CPB and after surgery associated with neurological damage and LCOS [133, 136].

##### Systemic Inflammation and Blood–Brain Barrier Compromise

While surgery by itself causes a systemic inflammatory response, exposure of blood cells to the circuit surface during CPB initiates an even stronger systemic inflammatory reaction, which worsens HI reperfusion injury [132, 137–139]. Infants with CHD have a preoperative increase in neuron-specific marker phosphorylated neurofilament heavy and the calcium-binding protein S100B, and detectable levels of neuron-specific enolase (NSE) in their plasma, which suggest a compromised blood–brain barrier. Infants with cyanotic CHD have higher plasma S100B levels preoperatively, during surgery with CPB and at 24 h postoperatively than infants with non-cyanotic CHD. The increases in perioperative plasma S100B levels correlated with increased oxygenation levels during CPB. This is thought to reflect hyperoxia stress during reperfusion***.*** Although S100B is not exclusively a neuronal marker, neuronal damage is the most likely source of elevated plasma S100B levels. Elevated S100B release from other sources such as adipose tissues is unlikely because the adipose marker adiponectin remains constant in the perioperative period [137, 140, 141]. CHD also increases serum levels of the complement proteins C5a and sC5b9 and the inflammatory cytokines IL-12p70, IL‑6, IL‑8, IL‑10 and TNF‑α levels preoperatively. CPB further increases cytokine levels and complement remains activated after surgery [137]. The use of modified ultrafiltration during CPB reduces inflammation and morbidity after surgery [132].

##### Systemic Lactate Levels

Lactate is formed during anaerobic metabolism. The greatest increase in lactate levels over the perioperative period occurs during CPB. Longer durations of CPB and aortic cross-clamping, low hematocrit levels during the operation, and younger ages at operation associated with increased postoperative serum lactate levels. Elevated postoperative serum lactate levels reflect reduced cerebral and renal oxygen saturation levels, which increases the risk of postoperative mortality and morbidity in infants [142–144].

##### Reoxygenation Injury and Controlled Oxygen Support During CPB

Standard CPB with a pump prime has a partial oxygen pressure that is relatively hyperoxic for a cyanotic patient. Cyanotic CHD patients, particularly those with the single-ventricle disease, have a high risk of reoxygenation and reperfusion injuries during CPB, which plays a central role inflammatory response induced by CPB. The plasma levels of the neuronal injury marker S100B increases as oxygenation increases during surgery. Controlling the partial oxygen pressure during CPB to match the patient’s partial oxygen levels reduced plasma markers of organ damage, inflammation, stress, and oxidative stress in single-ventricle patients, but not in double-ventricle patients [139, 140, 145, 146]. Supplementing nitric oxide (NO) has also been assessed as a strategy to reduce reperfusion injury. CPB damages endothelial cells which normally secrete NO. Hemoglobin released from hemolysis during CPB further scavenges NO. NO has a beneficial role in maintaining the vascular tone and reducing oxidative stress. Administration of NO donors during CPB has improved outcomes in pediatric trials [139].

#### Deep Hypothermic Circulatory Arrest (DHCA) and Continuous Hypothermic Low-Flow Bypass

The circulatory arrest is often used in open-heart surgery because of the absence of blood in the operative field. During this procedure, the body is cooled to 15–18 °C and the circulation is completely stopped, which induces a risk of hypoxic-ischemic and reperfusion brain injuries [73]. DHCA is based on the principle that there is a ‘safe’ duration for circulatory arrest under deep hypothermia [147]. However, extended durations of DHCA have deleterious effects on children. Traditionally periods of 45–50 min of DHCA have been considered safe [10]. However, the true ‘safe’ duration is likely much shorter. During the first 20 min of DHCA, the cerebral metabolism remains aerobic, after this period, anaerobic metabolism and cerebral lactate concentrations start to increase. Increased anaerobic metabolism eventually leads to cerebral energy failure [113]. The relationship between neurological impairment and the length of DHCA is not linear [10]. In HLHS children that had DHCA for the Norwood procedure, longer DHCA durations were associated with lower IQ scores in some, but not all, studies [10, 121]. Longer durations of hypothermia and support time and lower tympanic membrane temperatures during surgery were also associated with structural brain damage in d-TGA [37]. In general, the weight at operation and having DHCA were weak predictors of delays in PDI and MDI scores at 1–1.5 years of age in CHD, and the time of DHCA was a weak predictor of MDI scores. However, patient-specific factors were stronger predictors of neurodevelopmental outcomes than intraoperative factors [70].

Continuous hypothermic CPB at a low-flow rate (low-flow bypass) has been evaluated as an alternative to DHCA. The low-flow bypass has the advantages of maintaining the cerebral blood flow during surgery, but it requires longer durations of extracorporeal blood flow than DHCA, which increases the risk of pump-related brain injuries such as air or particulate emboli [10, 147]. A randomized trial which compared DHCA to low-flow bypass for d-TGA surgeries found that the EEG activity recovers more rapidly after low-flow bypass, which associates with fewer postoperative seizures and lowers brain creatinine kinase release [147].

#### Glucose Levels

Intraoperative hyperglycemia increases the risk of postoperative infections [148]. Infections may increase the postoperative length of stay, which is a risk factor for adverse neurodevelopmental outcomes [71]. However, intraoperative glucose levels were not correlated with long-term clinical, developmental or MRI deficits at 1, 4 or 8 years of age [149].

#### Anesthetic Use

The use of high doses of the benzodiazepine or volatile anesthetics in the perioperative and postoperative period associates with reduced neurodevelopmental scores at 1 year. A greater number of subsequent anesthetic exposures after the initial surgery was also associated with worse neurodevelopmental outcomes at 1 year of age [121, 126, 150]. At 1.5–2 years of age, there was no association between the dose and duration of anesthetic drugs in the perioperative period and on mental, motor and vocabulary scores [151]. At 4–5 years, there was a weak association between the days on chloral hydrate anesthesia during the perioperative period and lower performance IQ, the cumulative dose of benzodiazepine anesthesia and visual-motor integration scores, and the cumulative dose of volatile anesthetics and full-scale IQ and verbal IQ scores [152, 153].

### Postoperative Factors

Postoperative diffuse and focal WMI occurs in 26–50% of neonates with surgery for critical CHD. Hypoxemia and hypotension (both low mean and low diastolic blood pressure) in the early postoperative period, the age-at-operation and lower brain maturity scores associates with WMI [4, 16, 154]. Postoperative hyperglycemia increases the rate of early mortality and early CNS morbidity [155–157]. However, postoperative hyperglycemia was not associated with adverse neurodevelopmental outcomes at 1 year of age [158] and a randomized clinical trial of tight glycemic control postoperatively did not find improvements on mortality, morbidity or length of stay [159]. Postoperative cerebral desaturation due to low cardiac output or loss of cerebral autoregulation is common in infants with cardiac surgery for single-ventricle defects but it is unclear if cerebral desaturation predicts neurodevelopmental delays [160, 161]. The need for extracorporeal membrane oxygenation (ECMO) or ventricular assist device support postoperatively, as well as increases in the postoperative length of stay associated with lower scores on the PDI and MDI at 1–1.5 years of age [70, 71]. The use of ECMO and ventricular assist devices also increases the risk of stroke [115]. Additional postoperative risk factors for brain injuries in HLHS include multiple surgeries, prolonged hypoxemia, failure to thrive and the risk of strokes before and after the Fontan procedure due to abnormalities in coagulation factors [10]. Children with HLHS are operated at younger ages than children with other types of CHD and have a significantly longer postoperative length of stays, more surgeries and lower scores on tests of cognition, fine motor skills, executive function, and math skills at 4 years than infants than other subtypes of CHD. Children with d-TGA also have earlier surgeries with longer postoperative stays than infants operated for TOF or VSD. The neurodevelopmental performance at 4 years was comparable between d-TGA, TOF and VSD, but the children were more likely to have an impairment in at least 1 functional domain than the general population [162]. Additional reversible factors during development also play a role in neurodevelopmental outcomes. Obstructive sleep apnea occurred in 57% of a mild-moderate CHD cohort and was associated with worse neurodevelopmental outcomes [163]. Early motor screening and early physical therapy could alleviate some of the early locomotor deficits in CHD [164].

## Discussion

Neurological damage in CHD is a complex multifactorial insult affecting multiple brain regions. Here we discuss those innate patient factors, such as genetic risk factors, reduced cerebral blood flow and delayed brain growth, which results in an immature brain [16, 19, 20]. Adverse neurodevelopmental outcomes are likely due to the cumulative effect of critical CHD on several brain regions. CHD affects neural progenitor cells, which impairs the growth and maturation of the prefrontal cortex, which is essential for higher-order functions. Higher-order functions also require proper connectivity and communication between neurons throughout the brain. White matter development plays a crucial role in this and WMI and an underdeveloped white matter microstructure is frequently observed in CHD studies. Delayed development and injury of the subcortical areas, particularly the cerebellum, further impair neurocognitive performance [73–75, 83–85]. The immature brain is highly susceptible to hypoxia–hyperoxia and ischemia caused by cerebral blood flow disturbances due to the CHD itself or CPB during the surgical correction. An increased length of stay postoperatively also predicts worse outcomes, likely because it indicates a more complicated disease course [16, 19, 20].

Care must be taken when generalizing risk factors. CHD consists of a spectrum of disorders with varying degrees of severity and blood flow and blood oxygenation disruptions. Even after subdivision into syndromes such as d-TGA or HLHS, studies include heterogeneous patient populations with multiple risk factors for CNS dysmaturation and neurodevelopmental impairments. Neonates with multiple risk factors, such as genetic comorbidities, delayed brain growth, intraoperative and postoperative complications have a far greater risk of long-term disabilities than neonates with a prenatal diagnosis without additional comorbidities and a successful surgical repair [10]. These innate patient factors should be considered when predicting the prognosis for an individual patient and designing strategies to reduce neurological injuries in these populations.

There is a lot of room for improvement in understanding brain injuries after CHD. The current ‘best’ statistical models only explain around 30% of the variability in neurodevelopmental performance at 1 year of age and thus important factors remain unidentified [71]. There is also room for improving our outcome measures. The Bayley Scales of Infant Development is widely used to assess neurodevelopment within the first 3 years of life. However, a meta-analysis of the Bayley scales in preterm infants found that the MDI score explained 37% of the variance in cognitive functioning later in life, which was considered strongly predictive. The PDI scores only explained 12% of the variance and were considered moderately predictive. Thus, although the Bayley scales are helpful, they do account for the majority of variance in cognitive and motor performance later in life [165]. Having a low PDI or MDI score before 3 years does not necessarily mean the child will have poor performance at school age. The cognitive performance of children with low MDI can improve over time [166]. The reverse is also true. In a study of CHD children, there was an overall good correlation between scores on the Bayley scale at age 2 and neurodevelopmental performance at age 4. However, several children who scored in the average range of the Bayley scales at age 2, had developed neurodevelopmental deficits by age 4 [167]. Abnormal cerebral findings in the prenatal and preoperative period may be better predictors of neurodevelopmental deficits. Findings of a cerebral developmental delay, abnormal Doppler parameters of cerebral blood flow are particularly frequent in infants with a neurodevelopmental delay but no single cerebral abnormality predicts the poor neurodevelopmental outcome by itself. The neurodevelopmental outcome is likely the cumulative effects of delays in global brain development, combined with multiple hypoxic-ischemic events during the perinatal period, rather than a single factor [168].

Our current knowledge indicates that patient, preoperative and postoperative factors play a far greater role in adverse neurodevelopmental outcomes than perioperative techniques [71]. Unfortunately, innate patient factors are far more difficult to address and prevent than perioperative factors, and neurological damage starts early in gestation. Although cardiac surgery in the first few weeks of life can restore cardiac function, surgery does not correct pre-existing brain injuries and the brain developmental delays persist throughout life. Since the number of adults with CHD now outnumber children born with CHD, there is a need to identify ways to reduce WMI after birth and risk factors for secondary injuries across the lifespan [3, 6]. Successful mitigation of long-term neurological injuries will require consideration of the hemodynamic effects and brain injury patterns in different CHD types, more widespread adoption of fetal diagnosis and early surgical correction, and rehabilitation strategies to reduce functional deficits.

Understanding the impact of CHD on neurological functioning in adulthood and aging will be key in future studies. Data from the Quebec congenital heart disease database estimated the prevalence of CHD to be 8.23/1000 infants, 13.11/1000 children, 6.12/1000 adults and 3.80/1000 of the elderly. It is estimated that in 2010, there were 1.4 million adults and 1 million children living with CHD in the US [3]. Adult survivors of CHD have a high incidence of heart failure [169] and poor exercise capacity associated with a greater risk of hospitalization and death [170]. Higher plasma levels of von Willebrand factor, which reflect liver congestion due to right-sided heart failure, was also associated with increased all-cause mortality in adults with CHD [171]. A wide spectrum of structural brain abnormalities and lower estimated IQ levels has been identified in adults with CHD at a mean age of 26.7 years [128]. Further studies at more advanced ages are eagerly awaited.

There is an urgent need to discover therapies or rehabilitative strategies to improve neurological outcomes in CHD. This is further complicated by an incomplete understanding of the causes of brain dysmaturation in CHD. Continued research into animal and cellular models of CHD is essential to deliver on this need. It is now possible to develop cardiac and brain organoid systems for disease modeling and therapeutic discoveries [172]. Advances in CRISPR-cas9 gene-editing technologies allow us to improve our rodent, sheep and pig models of CHD. Combining our genetics, cell and molecular biology knowledge in future studies has great potential to find targets and therapies for neurological damage in CHD. Improvements to our clinical trial design are also essential to improve outcomes in CHD. The majority of clinical trials in CHD included heterogeneous cohorts with limited follow-up. Future prospective clinical trials should take care to include patients with the same type of CHD and combine this with meticulous long-term follow-up, such as in the Boston Circulatory Arrest Trial [74].

To conclude, we have made tremendous progress in improving the survival of infants with CHD over the past decades. Improving the quality of life of those survivors is our next frontier.
